# Dynamic economic emission dispatch of combined heat and power system based on multi-objective differential evolution algorithm

**DOI:** 10.1371/journal.pone.0326104

**Published:** 2025-06-13

**Authors:** Tao Dong

**Affiliations:** School of Economics and Trade, Henan University of Animal Husbandry and Economy, Zhengzhou, Henan, China; Aalto University, FINLAND

## Abstract

Engineering frequently deals with multi-objective optimization problems. In the scheduling of combined heat and power systems, the competing goals of economic cost and pollutant emission are challenging for conventional single-objective algorithms to handle, necessitating the use of effective multi-objective optimization algorithms. The research design improves the multi-objective differential evolution algorithm, which is constructed by making the scaling factor and crossover probability change adaptively, adopting non-dominated sorting, combining the congestion distance calculation to deal with multi-objectives, adding elite populations and quadratic mutation links, and so on. Based on this algorithm, the dynamic economic emission dispatch model of combined heat and power system is constructed to optimize the economic and environmental benefits of the system. The results revealed that the improved multi-objective differential evolution algorithm in Zitzler-Deb-Thiele 1 function test had generational distance of 0.0513, inverted generational distance of 0.3265, and hyper volume metric of 0.1301. Its Pareto optimal frontier fitted the standard curve better and was uniformly distributed, giving better performance. It was applied to solving dynamic economic emission dispatch model for combined heat and power system and compared with time-varying multi-objective PSO algorithm and others. Based on the ieee 30-node system deployment, it contained two cogeneration units, seven generator units, and one heating unit. The improved multi-objective differential evolution algorithm optimized the fuel cost as low as $2300590 and the pollution emission as low as 200285 kg. Its Pareto optimal frontier distribution was better, and it performed better in the hyper volume metric and inverted generational distance metrics. The research demonstrates that the improved multi-objective differential evolution algorithm can effectively balance operational cost and performance, achieving reduced fuel cost and pollution emissions. Furthermore, it exhibits strong adaptability and optimization capabilities in practical engineering applications, enhancing system operation efficiency and reducing pollution.

## 1. Introduction

In today’s evolving global energy landscape with stricter environmental protection, combined heat and power (CHP) systems, which are efficient for simultaneous generation of electricity and heat, are crucial for industrial production and district energy supply [1]. Existing research on CHP system scheduling has made great progress. Early static scheduling optimized energy distribution under fixed conditions and reduced economic costs [2]. As dynamic factors such as load and price fluctuations become more important, dynamic dispatch research using tools such as linear programming and genetic algorithms has emerged [3]. But current methods are limited. Most algorithms lack flexibility for complex dynamics and struggle with internal and external disturbances. To address this, it is important to introduce the multi-objective (MO) differential evolution algorithm (MDEA) to the dynamic economic emission dispatch (DEED) of CHP [4,5]. However, traditional MDEA can fall into local optima that do not meet the demands of modern energy control.

Multi-objective optimization (MOO) aims to balance multiple objectives, seeking trade-off solutions rather than single-objective optima. Li et al. built a MOO model and created an adaptive MDEA (AMDEA) based on deep reinforcement learning to address the scheduling issue in steel industry continuous annealing operations [6]. In comparison to existing MO evolutionary algorithms, the outcomes revealed that the algorithm could successfully direct the choice of variational operators and parameters and produce a better Pareto solution. Wang et al. proposed a diversity-based MDEA for feature selection (FS) by introducing a diversity score and a new binary variation operator to improve population diversity and FS performance [7]. This approach outperformed the already widely used MOFS techniques in FS, according to experiments conducted on 14 datasets. Du et al. considered the MO multi-task optimization problem as a MO multi-criteria (MC) optimization problem and proposed a framework of MOMC evolutionary algorithm to guide the individual selection and population evolution by integrating the evaluation criteria of multiple tasks [8]. Experiments verified the great effectiveness and efficiency of the proposed algorithm in solving MOMC optimization problems.Ali et al. number constraint technique and single and MO evolutionary algorithms to obtain better convergence and uniformly distributed Pareto frontiers [9].The outcomes indicated that the proposed method was able to find the near-global Pareto frontiers for highly complex problems while satisfying all operational constraints. Zhang et al. suggested a multi-modal and MO evolution algorithm (MMEA) based on independently evolving subproblems, which enhanced the diversity of the decision space through subproblem-independent evolution and two-phase environment selection strategy [10]. According to experimental data, the suggested algorithm outperformed seven other most advanced MMEAs in terms of competitive performance on two test function series.

CHP system scheduling refers to the process of achieving efficient energy utilization by optimizing the production and distribution of thermal and electrical energy. It ensures that energy consumption and environmental pollution are reduced while meeting the demand for electricity and heat by rationally adjusting the operating status of boilers, turbines, and other equipment. Sun et al. proposed a day-ahead scheduling strategy based on dynamic planning for optimizing a sustainable CHP system consisting of fuel cells, batteries, thermal energy storage, and heat pumps [11]. The optimization resulted in a highly coordinated and complementary operation of the system in terms of heat and power flow patterns over a 24-hour period, improving the overall efficiency by about 3.5% and facilitating the healthy operation of the batteries. Sun et al. employed a stochastic dynamic programming algorithm to deal with uncertainty in off-grid residential CHP systems and achieved globally optimal scheduling [12]. Simulation results indicated that the algorithm achieved efficient scheduling while improving the sustainability of the equipment and showed optimality and robustness under different levels of uncertainty. An enhanced modal factorization approach based on Q-learning was presented by Wang et al. for the low-carbon economic scheduling of CHP systems [13]. In addition to improving the wind power consumption space and system economics and lowering carbon emissions in simulation, the algorithm enhanced the search capabilities by dynamically modifying the crossover and variance probability.Baghaei et al. used optimal economic and emission dispatch of microgrids as an objective function (OF) and optimized it using MO genetic algorithm [14]. It was found that the profit could be significantly increased and the amount of pollution could be reduced by optimal solar panel tilt angle setting. Shukla and Pandit proposed a dynamic dispatch approach for CHP microgrids considering renewable energy sources and load uncertainty [15]. By integrating a fuzzy realization module and improving the differential evolution algorithm (DEA), the study achieved cost and emission reductions, as well as power loss reductions, both with and without considering storage.

Sundaram et al. proposed a dynamic economic emission scheduling optimization method that combined stochastic forest machine learning models to predict transmission losses, significantly reducing the computational burden and improving efficiency [16]. The research results showed that the proposed MO stochastic optimization algorithm could save fuel costs of $37,339.5 and $3,423.7 per year in the ieee 30 bus system compared to the existing algorithm. Sundaram proposed a new method for integrating artificial neural networks into dynamic economic emission scheduling models, which avoided the computational complexity of traditional methods by training neural networks to predict transmission losses [17]. The results showed that the algorithm could save US $ 92.92 in fuel costs and reduce 0.0375 tons of emissions per day in the Institute of Electrical and Electronics Engineers 30-bus system. Moreover, the computation speed was 30 times faster than non-dominated sorting genetic algorithm II and MO particle swarm optimization algorithm 7.6 times faster. Nalini et al. proposed improved single objective and MO algorithms based on the original moth flame optimizer, introduced the enhanced flame generation strategy into the MOO algorithm, combined with the crowding distance mechanism and non-dominated sorting framework, verified by 15 MO test functions, and introduced a new technique of incorporating loss prediction into the model with artificial neural network [18]. The results showed that the improved algorithm was superior to non-dominated sorting genetic algorithm, MO teaching-based optimization algorithm and MO reptile search algorithm in solving the dynamic economic emission scheduling problem of power system through the implementation on the 10-unit system and ieee 30 bus test system.

Chen et al. adopted multi-area combined heat and power economic dispatch (MRCHPED), integrated power-only units, cogeneration units and heat-only units, proposed an educational achievement guided group teaching optimization algorithm (EAGTOA), and introduced two historical archives to improve retrieval efficiency. The MRCHPED problems of 9 units in 2 regions, 17 units in 3 regions, and 96 units in 3 regions were solved. Simulation results showed that the EAGTOA algorithm had a good effect on the accuracy and convergence rate of the MRCHPED problem, which was superior to several classical and recent optimization algorithms [19]. Chen et al. proposed a derivative search-based policy optimization algorithm (DSPO) to solve the multi-regional economic scheduling (MAED) problem involving renewable energy with poor accuracy and slow convergence speed, and used Weibull and lognormal probability density functions to model wind and solar energy. Two strategies, leader guidance and derivative search, were used to improve the search performance and solve four types of MAED problems considering the valve point effect and other factors. Simulation showed that compared with existing algorithms, DSPO achieved better overall results in terms of convergence speed, solution accuracy, and stability [20]. Chen et al. sought to address the PV parameter estimation (PVPE) problem in solar photovoltaic system design. They proposed an improved synchronous heat transfer search (ISHTS) meta-heuristic algorithm to enhance search performance by integrating synchronous heat transfer and elite perturbation strategies, given the complexity of PV models. The ISHTS algorithm was employed to address a range of PVPE challenges, including single diode problems. Simulation showed that for the four PV models, the root mean square error of ISHTS was as low as 9.8248 × 10−4, and the standard deviation was as small as 8.88 × 10−17, which had significant advantages in solving accuracy, reliability and convergence [21].

In CHP system scheduling, researchers have proposed optimization algorithms to improve system efficiency and reduce energy consumption and emissions. However, some algorithms lack flexibility and real-time performance in complex real-world conditions. In DEED research, algorithms such as the combination of random forests and neural networks show good results in cost and emission reduction, but need improvement in universality and grid integration. Research on MOO problems has made remarkable progress, providing novel concepts and technical means. However, in CHP scheduling, existing optimization strategies have drawbacks. Some researchers fail to balance MOO objectives, resulting in an uneven Pareto front. Moreover, certain algorithms perform poorly in large scale, high dimensional problems, which affects optimization efficiency. To address these issues, this study proposes CHP dynamic economic emission dispatch (CHPDEED). It considers the MO characteristics of CHP systems and balances economic and emission objectives through adaptive operator and parameter adjustment. Compared with existing methods, MDEA exhibits better convergence and Pareto frontier distribution in dispatching CHP production systems, facilitating efficient energy utilization and pollution reduction.

The novelty of this study is significant. In the context of addressing challenges associated with MOO problems, the conventional approach is characterized by numerous limitations. These limitations include the fixed scaling factor and crossover probability in the standard DEA algorithm, which impacts the optimization search efficiency. Additionally, the subjectivity of weight setting and the complexity of obtaining the complete Pareto frontier in the original weighted summation processing of MO are frequently encountered. This study improves the search efficiency of the algorithm by making scaling factor and crossover probability adapt to change. Concurrently, the integration of non-dominant sorting and crowded distance calculation effectively overcomes the limitations of traditional weighted summation methods. This approach enables the successful extraction of the entire Pareto frontier, thereby addressing the research gaps identified in previous studies.

The contribution of this study is that it adaptively adjusts the scaling factor and crossover probability by proposing an improved MDEA that sets the elite group and introduces quadratic variation. This can solve the problem that the traditional DEA is inefficient and easy to fall into local optimum in MOO. By constructing the dynamic economic emission scheduling model of CHP system, the improved MDEA is applied to solve the MOO problem of how to minimize fuel cost and pollutant emission when CHP system meets the demand of electricity and heat load. Furthermore, it provides a more efficient and reliable solution to the MOO problem in the engineering field.

There are four sections to the study. An overview of the pertinent background and research is given in the first section. The second section is to improve the MDEA and construct the CHPDEED model. Verifying and evaluating the algorithm’s and model’s performance is the third section. The entire research is summarized in the fourth section.

## 2. Methods and materials

### 2.1. Improved mdea design

**Fig 1:**
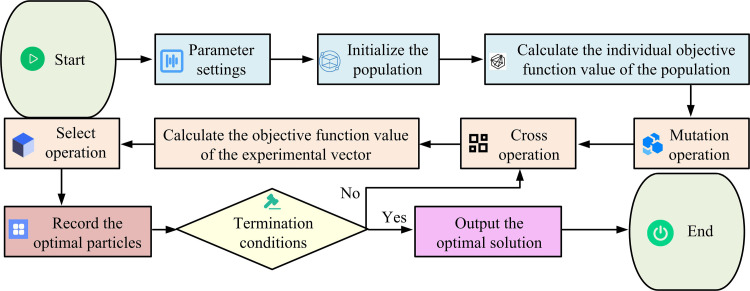
DEA process.

MOO problems are common in engineering. For example, CHP scheduling involves conflicting economic and emissions objectives. Traditional single objective algorithms fall short. DEA, an effective MOO algorithm, finds balanced trade-off solutions via population evolution. The DEA process is shown in Fig 1.

In Fig 1, randomly generating the initial population is a key step in the startup of the algorithm. It is assumed N that the population size is N, the individual representation is $X_{i}^{0}$, and the individual dimension is D. The initial population generation formula is shown in Equation (1). The initialization method of this study is to build a formula based on population size, individual dimension, and other parameters, and generate initial populations randomly based on uniform distribution. Unlike initialization based on chaos or opposites, it does not rely on chaotic properties or opposites, but introduces diversity directly into the population.


$$X_{ij}^{0}=LB_{j}+rand(0,1)\cdot (UB_{j}-LB_{j}),j\in 1,2,...,D$$ (1)

In Equation (1), the decision variable of the j dimension of the i individual is $X_{ij}^{0}$, and the initial population is $X_{ij}^{0}$. The upper and lower limits (ULL) of the j th variable are $UB_{j}$ and $LB_{j}$. The random generating function with a uniform distribution between 0 and 1 is rand(0,1). The variance operation aims to introduce population diversity, which is the core aspect of differential evolution. The formula for generating the variation vector is shown in Equation (2).


$$V_{i}^{t}=X_{r1}^{t}+F\times (X_{r2}^{t}-X_{r3}^{t})$$ (2)

In Equation (2), the variation vector of the target individual $X_{i}^{t}$ is $V_{i}^{t}$. The current iteration number is t. The three mutually identical indexes are r1, r2, and r3, respectively. The scaling factor is F, which controls the scaling degree of the difference vector, determines the magnitude of variation, and helps the algorithm to jump out of the local optimum. The crossover operation is used to combine the variance vectors with the information of the original individual to generate the test individual. The binomial crossover rule is shown in Equation (3).


$$U_{ij}^{t}=\{\begin{array}{c} {}*20cV_{ij}^{t},if\;rand(0,1)\leq CR\;or\;j=j_{rand}\\ X_{ij}^{t},\;\;\;\;\;\;\;\;\;\;\;\;\;\;\;\;\;\;\;\;\;\;\;\;\;\;otherwise \end{array}$$ (3)

In Equation (3), the test individual is $U_{ij}^{t}$. The crossover probability is CR. The dimension index randomly selected from 1 to D is $j_{rand}$. It is ensured that at least one dimension of the test individual comes from the variation vector, which promotes the new individual to integrate the changes brought by the variation with the original individual’s good characteristics. Selection is made based on the greedy strategy, comparing the fitness values of the test individuals and the parent individuals to determine the next-generation parent individuals. Equation (4) illustrates how the integrated fitness function is constructed using the weighted summation method.


$$Fit(X)=\omega_{1}f_{1}(X)+\omega_{2}f_{2}(X)$$ (4)

In Equation (4), the integrated fitness value is Fit(X). The two OFs are $f_{1}$ and $f_{2}$, respectively. The weighting coefficients are $\omega_{1}$ and $\omega_{2}$, respectively. In Equation (4), the weighting function is used to combine the cost and emission and other objectives and convert them into a single scalar function. It seems to weaken the uniqueness of the multi-objective algorithm. However, in fact, this method is still of great significance in MOO. By reasonably setting the weight coefficient, the conflicts between different objectives can be effectively balanced. In addition, combined with operations such as non-dominated sorting and crowded distance calculation, the advantages of multi-objective algorithm in finding approximate Pareto frontier solutions can be fully utilized to provide an implementable solution. When determining the values of $\omega_{1}$ and $\omega_{2}$, it is necessary to determine the importance of the specific optimization objective. If the cost objective is more critical, the weight of $\omega_{1}$ can be appropriately increased. If the emission target has a high priority, the proportion of $\omega_{2}$ should be increased. Reasonable setting of the two values can effectively balance the conflict between cost and emission targets. In the optimization process of objective minimization, the selection rule of the next generation of parent individuals is shown in Equation (5).


$$X_{i}^{t+1}=\{\begin{array}{c} {}*20cU_{i}^{t},Fit(U_{i}^{t})\leq Fit(X_{i}^{t})\\ X_{i}^{t},\;\;\;\;\;\;\;\;\;\;\;\;otherwise \end{array}$$ (5)

In Equation (5), the next-generation parent individual is $X_{i}^{t+1}$. Boundary processing is critical to keeping parameters feasible. The random replacement method regenerates overboundary values, while the boundary absorption method sets them to boundary values to ensure algorithm stability. The scaling factorF and crossover probability CR of the conventional DEA are fixed, limiting the efficiency of optimization search, and the improvement idea of the study is to make them change adaptively. The dynamic scaling factor is shown in Equation (6).


$$F_{i}^{t}=F_{\min}+(F_{\max}-F_{\min})\times \frac{e^{1-\frac{Gen_{\max}-t}{Gen_{\max}}}-e^{-1}}{e-e^{-1}}$$ (6)

In Equation (6), the ULL of the scaling factor values are $F_{\max}$ and $F_{\min}$. The maximum number of iterations is $Gen_{\max}$. The cross-probability adaptive adjustment formula is shown in Equation (7).


$$CR_{i}^{t}=CR_{\min}+(CR_{\max}-CR_{\min})\times \frac{t}{Gen_{\max}}$$ (7)

**Fig 2:**
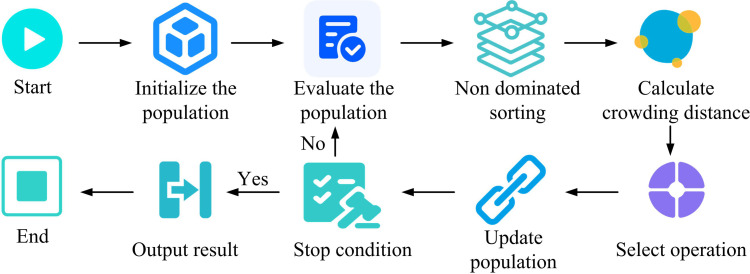
Steps for obtaining a complete Pareto frontier.

In Equation (7), the ULL of the crossover probability are $CR_{\max}$ and $CR_{\min}$. The probability of crossover increases during iteration. It retains more original genes early and encourages mutated genes to merge later for a balanced search. An elite population is created to avoid losing good individuals. After each generation it is compared with the new population. Inferior individuals in the new generation are replaced to ensure evolution [22]. The elite population plays the role of storing the best individuals in the population in the algorithm. Moreover, the individuals with the best fitness are selected to be stored in the elite population in each generation. After the selection operation, the new generation population is compared with the elite population, if there is a better individual in the elite population, the worst individual in the new generation population is replaced with it. This mechanism avoids the loss of good individuals due to randomness in the process of evolution and ensures the continuous evolution of the population in a better direction. At the same time, individuals in the elite group provide guidance for the algorithm search, which promotes the algorithm to jump out of the local optimum and enhances the exploration of the solution space. This can effectively avoid algorithm stagnation and improve the algorithm’s solution performance in MOO problems. In practical applications, operators have an urgent need for implementable solutions from the Pareto frontier. At present, the extant techniques for selecting the optimal compromise solution encompass weight-based approaches, which amalgamate considerations to obtain a compromise by assigning weights to disparate objectives. There are also goal-based sorting methods that filter according to the importance of the goals. In this study, a combination of non-dominant sorting and crowding distance calculation is used. The non-dominant order divides the population into different levels of non-dominant layers, and the crowding distance measures the crowding around an individual. According to the principle of priority of non-dominant layer and large priority of congestion distance in the same layer, the algorithm can be effectively guided to find an approximate solution to optimize the complete Pareto frontier. This, in turn, it provides an implementable solution for operators. The steps to obtain the complete Pareto frontier are shown in Fig 2.

The non-dominated ordering divides the population into different levels of non-dominated tiers. Tier 1 is the set of non-dominated individuals in the current population. After removing these individuals, the remaining individuals then find the non-dominated set to form tier 2, and so on. Crowding distance measures the degree of crowding around an individual, and a large crowding distance results in a sparse solution around the individual, which is more valuable for retention. The calculation is shown in Equation (8).


$$CD_{i}=\sum_{k=1}^{m}\frac{\Delta f_{ki}+\Delta f_{k(i+1)}}{f_{k}^{\max}-f_{k}^{\min}}$$ (8)

**Fig 3:**
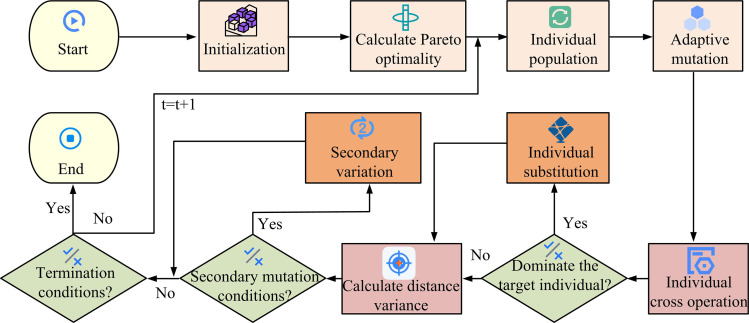
Improvements to the MDEA process.

In Equation (8), the quantity of OFs is m. The maximum value (MaxV) and minimum value (MinV) of the k th OF $f_{k}$ in the current population are $f_{k}^{\max}$ and $f_{k}^{\min}$, respectively. The selection operation is based on the principles of non-dominated layer hierarchy priority and the same layer according to the principle of large congestion distance priority to ensure that the algorithm seeks to optimize the approximation of the complete Pareto frontier. Finally, the DEA with DE/best/1 mutation strategy is studied, which converges quickly but is prone to local optimization. For this reason, a quadratic variation link is added after the selection operation to determine whether the variables are clustered using the optimized variable distance variance formula. If the variance is less than 0.1, the variables are adjusted according to the stochastic perturbation formula, and the search is restarted to prevent falling into local optimality. At this point, the improved MDEA construction is completed. Its flow is shown in Fig 3.

In Fig 3, after starting the MDEA, the initial population is generated by initializing and entering the algorithm parameters. The Pareto optimality of the initial population is then computed and the results are applied to the external population. Adaptive mutation is then performed on individuals in the population, introducing new traits by changing individual genes [23]. Individual crossover is then performed to combine the genes of different individuals. After the operation is completed, it is determined whether the experimental individual dominates the target individual, and if so, individual replacement is performed. If not, the distance variance is calculated for all individuals in the population. It then determines whether the quadratic mutation condition is satisfied and performs the quadratic mutation operation to further explore the search space. The above process is repeated continuously, increasing the value of each iteration by 1, until the termination condition is satisfied, at which point the algorithm is terminated.

### 2.2. Deed model construction for chp systems

**Fig 4:**
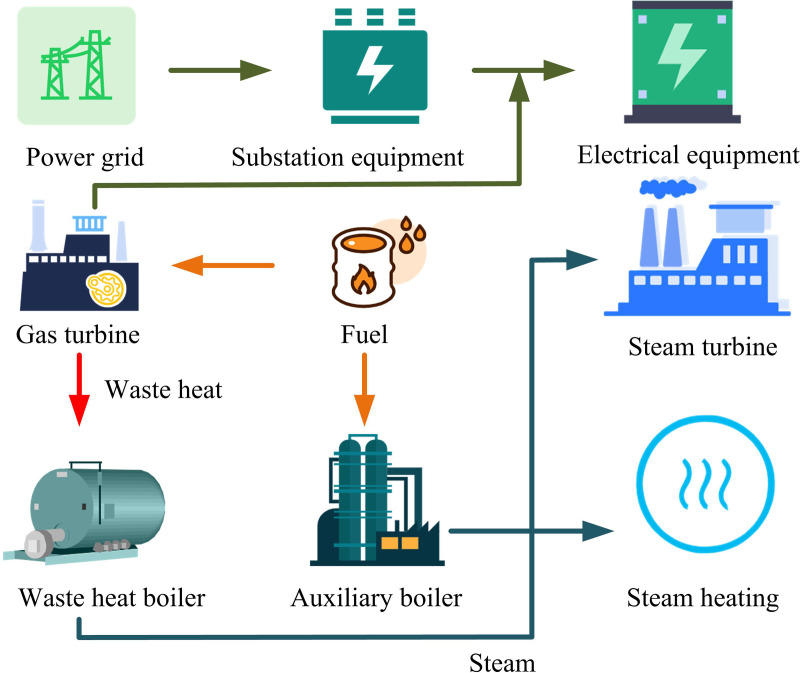
CHP system architecture.

The improved MDEA provides an algorithmic basis for DEED of CHP system. Based on this foundation, the study will further construct the CHPDEED model to achieve efficient optimization of system operation. The CHP system structure is shown in Figure 4.

The power unit and the heating apparatus are the key components of the CHP system, as shown in Fig 4. The gas turbine and steam turbine in the power unit are in charge of transforming fuel energy into mechanical energy so that power can be produced [24]. The waste heat generated from power generation is recovered with the help of heat supply components such as waste heat boilers and heat exchangers. The heat energy is then precisely deployed through pipelines and valves to finally reach the synchronized output of heat and power. It utilizes energy efficiently to meet the multiple demands of electricity and heat. DEED takes into account the operation of the system at different time stages to optimize the economic and environmental benefits [25]. To simplify the analysis, it is assumed that fuel prices remain stable over the planning period and their fluctuations are not taken into account. This assumption is valid for periods when fuel market prices are relatively stable, but the limitation is that when there are large fluctuations in the market, the model forecast may differ from the actual situation. The selection of emission factors is consistent with the industry’s general standards, and its applicability is evident in its capacity to reflect the relationship between pollutant emission and power generation or heat supply under normal circumstances. However, it should be noted that the actual emission situation of different regions and different equipment may vary. In addition, the model does not take into account the effect of equipment aging, which is appropriate for the stage when the equipment is newly commissioned or the degree of aging is slight. With the aging of the equipment, the situation such as the decrease of the equipment efficiency and the increase of the failure rate is not reflected in the model. For transmission loss, the model sets a fixed loss rate. It has a certain applicability when the system operating conditions are relatively stable. However, the actual transmission loss is affected by many factors such as load changes and line aging. This assumption cannot accurately reflect these complex changes. The CHP-DEED model is suitable for the scenarios where fuel price is stable, the equipment is newly put into operation or the aging degree is light, and the transmission loss is relatively fixed. It can make efficient use of energy and meet the multiple demands of power and heat simultaneously. However, its limitations are that when fuel prices fluctuate significantly, equipment aging leads to decreased efficiency or increased failure rates, actual emissions and industry standards differ greatly, and transmission losses are affected by load changes or line aging, the model’s prediction results may be biased from the actual situation, and cannot accurately reflect the system operating status under complex conditions. Therefore, the system needs to be adjusted and optimized in practical application according to specific engineering conditions. The main objective of CHPDEED model is to minimize the fuel cost (FC) and pollutant emissions while meeting the demand of electricity and heat loads. The FC calculation is shown in Equation (9).


$$C_{total}=C_{gen}+C_{chp}+C_{heat}$$ (9)

In Equation (9), the total FC is $C_{total}$. The FC of the generating unit is $C_{gen}$. The FC of the CHP unit is $C_{chp}$. The fuel of the heat generating unit is $C_{heat}$. The pollution emission is calculated as shown in Equation (10).


$$E_{total}=E_{gen}+E_{chp}+E_{heat}$$ (10)

In Equation (10), the total pollutant emissions are $E_{total}$. The pollutant gas emissions from the generating unit are $E_{gen}$. The pollutant gas emissions from the CHP unit are $E_{chp}$. The pollutant gas emissions from the heat generating unit are $E_{heat}$. The electrical power (EP) balance constraint is shown in Equation (11).


$$\sum_{i\in gen}P_{gen,i}+\sum_{j\in chp}P_{chp,j}=P_{load}$$ (11)

In Equation (11), the EP of the i th generating unit is $P_{gen,i}$. The EP of the j th CHP unit is $P_{chp,j}$. The total electrical load demand of the system is $P_{load}$. The thermal power (TP) balance constraint is shown in Equation (12).


$$\sum_{j\in chp}Q_{gen,i}+\sum_{k\in heat}Q_{heat,j}=Q_{load}$$ (12)

In Equation (12), the TP of the j th CHP unit is $Q_{chp,i}$. The TP of the k th heat generating unit is $Q_{heat,j}$. The total thermal load demand of the system is $Q_{load}$. The power constraint of the generating unit is shown in Equation (13).


$$P_{gen,\min,i}\leq P_{gen,i}\leq P_{gen,\max,i}$$ (13)

In Equation (13), the ULL on the power of the i th generating unit are $P_{gen,\max,i}$ and $P_{gen,\min,i}$, respectively. The power constraints of the CHP unit are shown in Equation (14).


$$\left\{ \begin{array}{c} P_{chp,\min,j}\leq P_{chp,j}\leq P_{chp,\max,j}\\ Q_{gen,\min,j}\leq Q_{gen,j}\leq Q_{gen,\max,j} \end{array}\right.$$ (14)

In Equation (14), the ULL on the EP of the j th CHP unit are $P_{chp,\max,j}$ and $P_{chp,\min,j}$, respectively. The ULL on its TP are $Q_{gen,\max,j}$ and $Q_{gen,\min,j}$, respectively. The power constraints of the heating unit are shown in Equation (15).


$$Q_{heat,\min,k}\leq Q_{heat,k}\leq Q_{heat,\max,k}$$ (15)

In Equation (15), the ULL on the TP of the k th heating unit are $Q_{heat,\max,k}$ and $Q_{heat,\min,k}$, respectively. There are four decision-making variables of the system, $P_{gen,i}$ can change the total amount of system power generation by adjusting the variable. $P_{chp,j}$ not only affects the system power supply, but also is related to the overall operating state of the CHP unit. $Q_{chp,i}$ determines the heating capacity of the CHP unit and its contribution to the system heat supply. $Q_{heat,j}$ is directly related to the system heat supply. These decision variables are interrelated and restricted, and together constitute the decision variable vector. It has an important impact on the operation cost, energy supply balance and pollutant emission of the entire cogeneration system. By reasonable value and optimization of them, the system can achieve optimal operation under multi-objective constraints. The unit climb rate constraints are shown in Equation (16).


$$-R_{down}\leq P_{t+1}-P_{t}\leq R_{up}$$ (16)

In Equation (16), the climbing rates in adjacent time periods are $P_{t}$ and $P_{t+1}$, respectively. The downward climbing rate of the unit is constrained to be $R_{down}$. The upward climbing rate is constrained to be $R_{up}$. In the improved MDEA for solving the CHPDEED problem, the algorithm first considers each individual in the population as a potential solution to the problem to form the Pareto-optimal set of solutions to the problem. Constraint handling is the key aspect, and the algorithm ensures that the individuals satisfy the constraints on the electrical and TP balance, the power of the generating unit, the CHP unit, and the rate of uphill climb by means of a specific repair strategy [26–28]. When a constraint violation occurs, the algorithm adjusts the unit output according to the constraints until the condition is satisfied. Furthermore, the calculation of the constraint violation value enables the algorithm to evaluate the feasibility of the resulting solution. This assessment ensures that the solution is both effective and consistent with the constraint requirements of the underlying problem [29].The constraint violation value calculation formula is shown in Equation (17).


$$V_{total}=V_{ep}+V_{hp}+V_{gen}+V_{chp}+V_{heat}$$ (17)

In Equation (17), the total constraint violation value is $V_{total}$. The electrical energy constraint violation value is $V_{ep}$. The thermal energy constraint violation value is $V_{hp}$. The constraint violation values for the generator unit, CHP unit, and thermoelectric unit are $V_{gen}$, $V_{chp}$, and $V_{heat}$, respectively. The expression for the constraint violation value is shown in Equation (18).


$$\left\{ \begin{array}{c} V_{ep}=\left|\sum_{i}P_{gen,i}+\sum_{i}P_{chp,j}-P_{demand}\right|\\ V_{hp}=\left|\sum_{i}Q_{gen,i}+\sum_{i}Q_{heat,k}-Q_{demand}\right|\\ V_{gen}=\max(0,P_{gen,\max}-P_{gen,actual})\\ \;\;\;\;\;\;\;\;+\max(0,P_{gen,actual}-P_{gen,\min})\\ V_{chp}=\max(0,P_{chp,\max}-P_{chp,actual})\\ \;\;\;\;\;\;\;\;\;+\max(0,P_{chp,actual}-P_{chp,\min})\\ \;\;\;\;\;\;\;\;\;+\max(0,Q_{chp,\max}-Q_{chp,actual})\\ \;\;\;\;\;\;\;\;\;+\max(0,Q_{chp,actual}-Q_{chp,\min})\\ V_{heat}=\max(0,Q_{heat,\max}-Q_{heat,actual})\\ \;\;\;\;\;\;\;\;\;+\max(0,Q_{heat,actual}-Q_{heat,\min}) \end{array}\right.$$ (18)

In Equation (18), the electric energy demand value is $P_{demand}$. The thermal energy demand value is $Q_{demand}$. The actual generation value of the generator set and CHP unit is $P_{gen,actual}$ and $P_{chp,actual}$ respectively. The actual output value of the CHP unit and the thermoelectric unit is $Q_{chp,actual}$ and $Q_{heat,actual}$, respectively.

**Fig 5:**
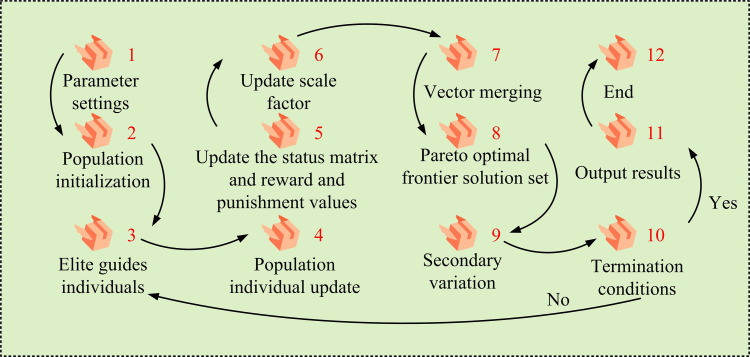
Steps for solving the DEED problem for the CHP system.

Constraint processing is critical to solving the CHPDEED problem with improved MDEA. The algorithm treats each individual population as a potential solution. Repair strategies ensure that it satisfies the power and thermal balance, generation unit power, CHP unit power, and climb rate constraints. When a violation occurs, the algorithm adjusts the unit output until the constraints are satisfied. By calculating the violation value, it evaluates the feasibility of the solution. In the solution process, constraint processing modifies individuals during generation. Later, during population update, it constrains the trial vector and calculates relevant indicators. Through iterative optimization, the final solution satisfies all constraints [30]. The steps for solving the CHPDEED problem are shown in Fig 5.

Improved MDEA first generates individuals with initialized populations for systems containing generating units, CHP units, and heat generating units. Then, the individuals are modified by constraint processing technique to calculate the target value and constraint violation value. After the above operations, the individuals are ranked by non-dominated sorting and congestion distance calculation, and the top 10% elite individuals are selected for bootstrapping [31,32]. In the population renewal phase, the elite bootstrap variation strategy is applied to generate variation, experimental vectors. The experimental vector constraints are processed and their fuel expenses, pollutant gas emissions are calculated. Whereas, it compares the dominance relationship between experimental vectors and target vectors, updates the state matrix, reward and penalty values, and then updates the scaling factor. The two types of vectors are merged and non-dominated sorting, congestion distance calculation is done again. The first N individuals are selected to form a new target vector, which is recorded as the Pareto optimal frontier (POF) solution set. According to the distance variance, it is judged whether the quadratic variation is performed or not. Finally, if the termination condition is not met, return to repeat the iteration. When satisfied, the solution set and OF value are output to complete the solution [33,34].

When evaluating constraints and objectives, an initial population of potential solutions is randomly generated. First, the power balance is checked: the sum of generator set and CHP unit power is compared to the system power load. If insufficient, units are adjusted. Similarly, for the thermal balance, the CHP and heating unit outputs are compared with the thermal load requirements and adjusted as necessary. Each unit’s power is verified to be within limits. The fuel cost and pollutant emissions of each potential solution are calculated. For the climb rate constraint, climb rates in adjacent periods are compared to limits and adjusted. Through iterative optimization, solutions balance cost and environmental objectives under various constraints, and the optimal solution is selected.

## 3. Results

### 3.1. Improved mdea performance analysis

In the experimental environment configuration, the hardware platform uses Intel Core i7 processor with 16GB RAM and NVIDIA GeForce RTX 3060 graphics card. The software environment is based on Python 3.8. The development environment selects PyCharm Professional Edition, which supports Python code writing, debugging and performance analysis, and also integrates version control tools. To speed up the execution of the algorithm, the multiprocessing library is used for parallel computation in the experiments. The simulation and testing of the algorithms are performed in Jupyter Notebook. The experiment compares MDEA, AMDEA, and non-dominated sorting genetic algorithm II (NSGA-II) in order to validate the performance of the enhanced MDEA. Moreover, the Zitzler-Deb-Thiele (ZDT) test function set is used for testing. Among the parameter settings, the number of iterations is 150 and 300. The initial value of crossover probability is set to 0.75, because near this value, the algorithm can better balance the fusion of original gene and variant gene. Therefore, the population retains more excellent characteristics of the original in the first iteration. With the progress of iteration, the crossover probability of adaptive adjustment can increase the fusion of variant gene and balance the global and local search ability. The value range of the scaling factor is set to [0.5, 0.8], within which the scaling factor can effectively control the degree of scaling of the difference vector and help the algorithm jump out of the local optimum. The sensitivity analysis mainly involves crossover probability and scaling factor, convergence speed, Pareto frontier uniformity and optimal solution objective function. By adjusting the combination of these parameters, the effect on the performance of the algorithm is analyzed, and the optimal parameter configuration is determined. The results of parameter sensitivity analysis are shown in Table 1.

**Table 1:** Parameter sensitivity analysis results.

Crossover probability	Scaling factor	Convergence iterations	Pareto frontier uniformity	Optimal value
0.6	0.5	120	0.35	282
0.6	0.6	100	0.27	261
0.6	0.8	90	0.32	273
0.7	0.5	110	0.25	256
0.7	0.6	80	0.21	232
0.7	0.8	75	0.22	247
0.8	0.5	130	0.27	265
0.8	0.6	70	0.18	221
0.8	0.8	65	0.23	236

**Fig 6:**
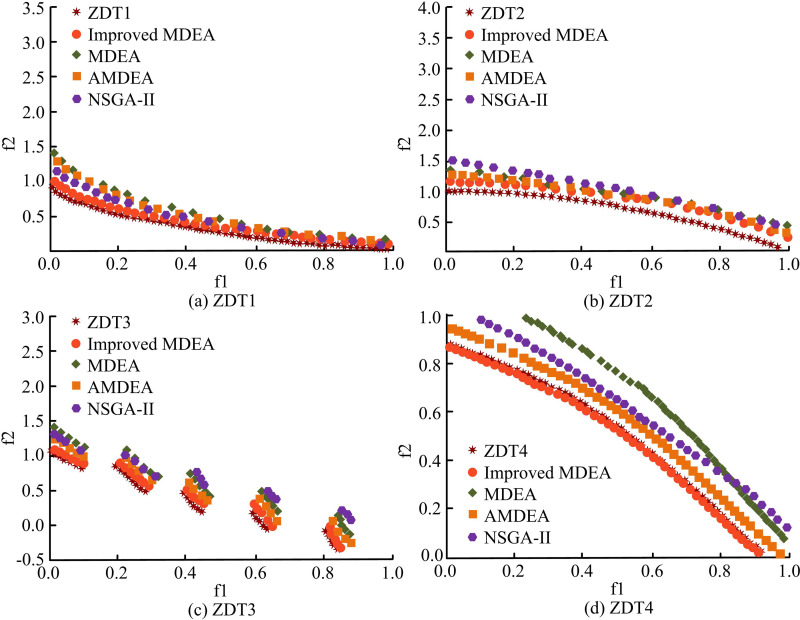
POF after 150 iterations.

In terms of convergence speed, when the crossover probability is 0.7 or 0.8 and the scaling factor is 0.6, the number of iterations is relatively small and the convergence speed is faster. For example, when the crossover probability is 0.7 and the scaling factor is 0.8, the number of iterations is only 75 times. It indicates that the algorithm can quickly find a satisfactory solution. In terms of Pareto frontier uniformity, when the crossover probability is 0.8 and the scaling factor is 0.6, the average distance between adjacent points is the smallest (0.18). It indicates that the Pareto frontier uniformity is the best and the solution distribution is more reasonable. In terms of the OF value of the optimal solution, when the crossover probability is 0.8 and the scaling factor is 0.6, the OF value is the smallest (220). It means that the solution obtained under this parameter combination is optimal in terms of comprehensive cost. In summary, an analysis of the performance data of algorithms with different combinations of crossover probability and scaling factor reveals that the improved MDEA demonstrates superior performance in convergence speed, Pareto frontier uniformity, and the OF value of the optimal solution when the crossover probability is set at 0.8 and the scaling factor is 0.6. The POF after 150 iterations is shown in Fig 6.

**Fig 7:**
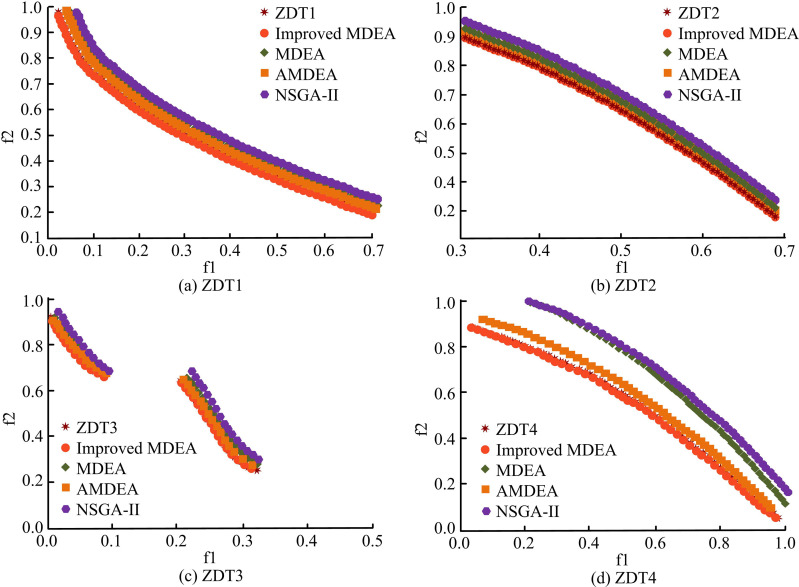
POF after 300 iterations.

Fig 6(a),6(b),6(c), and 6(d) shows the frontier plot for the ZDT1, ZDT2, ZDT3, and ZDT4 function test. The quantity of iterations of the algorithm is less and the fit to the standard curve is lower. Compared to MDEA, AMDEA, and NSGA-II, the POF of the studied improved MDEA has a better fit to the standard curve and a more uniform distribution. Fig 7 displays the POF after 300 iterations.

**Fig 8:**
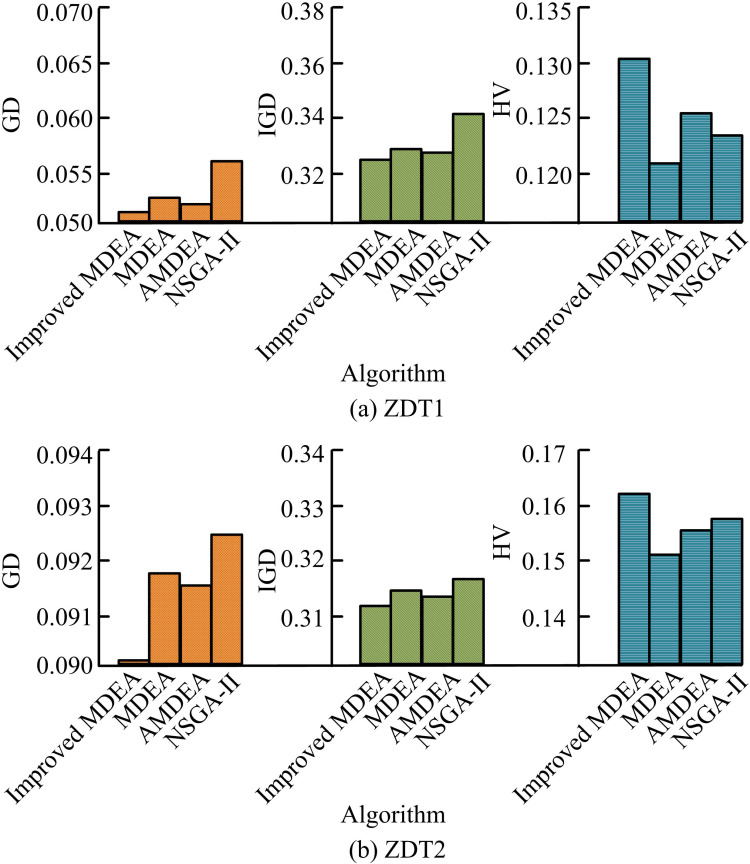
Comparison of algorithm performance in ZDT1 and ZDT2 function tests.

In Fig 7, when the iteration is increased, the fit of the algorithm’s POF to the standard curve is further improved. Some algorithms may not converge to the optimal solution within a given number of iterations. However, with the increase of iterations, the algorithm gradually approaches to find a more accurate POF. Therefore, it can be understood that the existing algorithm is able to find the POF. However, the performance and efficiency increase with the increase of the number of iterations. Among the four algorithms, the POF of the studied improved MDEA fits the standard curve more closely and the particles are uniformly distributed. This suggests that the enhanced MDEA’s optimization effect outperforms the comparison algorithms in a range of difficult function scenarios and exhibits strong performance and flexibility. The experiment use the hyper volume (HV) metric to measure the algorithm’s diversity and rate of convergence in order to quantitatively assess its effectiveness. Higher values denote greater performance. The inverted generational distance (IGD) metric measures how close the non-dominated solution (NDS) set is to the true optimal frontier as well as the uniformity and diversity of the population. The convergence of the NDS set is assessed using the generational distance (GD) measure. Lower values of GD and IGD suggest that the method is performing better. The performance comparison of the algorithms in the ZDT1 and ZDT2 function tests is shown in Fig 8.

**Fig 9:**
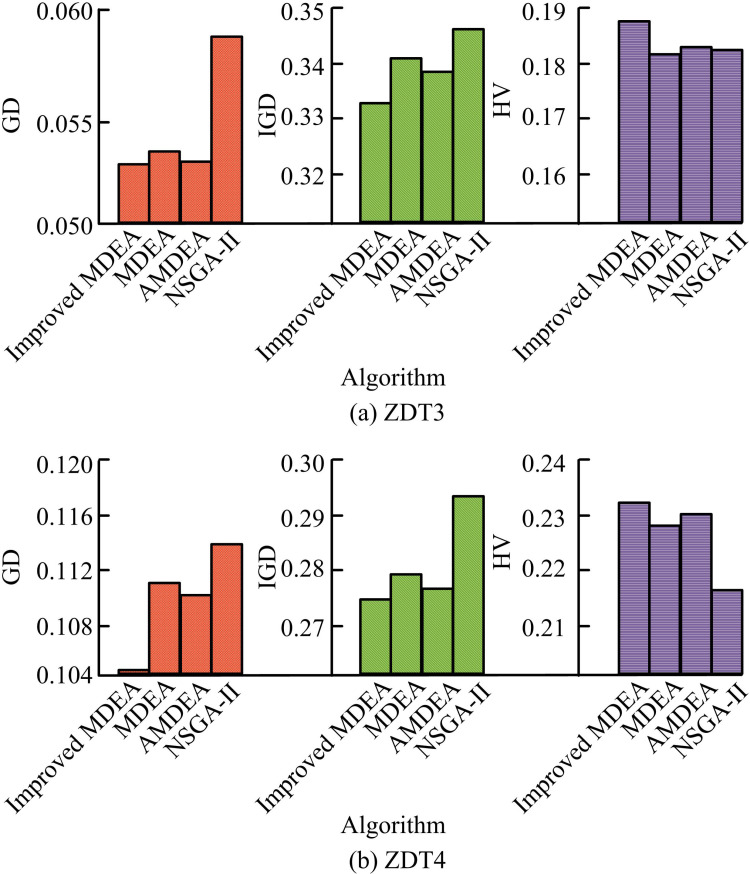
Comparison of algorithm performance in ZDT3 and ZDT4 function tests.

Fig 8(a) shows the performance comparison of ZDT1 function test, the GD and IGD of improved MDEA are 0.0513 and 0.3265, which are lower than MDEA, AMDEA, and NSGA-II algorithms. The HV of the improved MDEA is 0.1301, which is higher than the 0.1239 of the NSGA-II algorithm. Fig 8(b) shows the performance comparison of the ZDT2 function test, and the GD of the improved MDEA is 0.0903, which is lower than that of AMDEA’s 0.0916. The IGD of the improved MDEA is 0.3126, which is lower than that of AMDEA’s 0.3135. The HV of the improved MDEA is 0.1634, which is higher than the 0.1578 of the NSGA-II algorithm. The performance comparison of the algorithms in the ZDT3 and ZDT4 function tests is shown in Fig 9.

Fig 9(a) displays the performance comparison of the ZDT3 function test. Among the four algorithms, improved MDEA has the highest HV of 0.1884. Fig 9(b) shows the performance comparison of the ZDT4 function test. The GD and IGD of improved MDEA are 0.1048 and 0.2789, which are lower than MDEA, AMDEA, and NSGA-II algorithms. The HV of the improved MDEA is 0.2316, which is higher than 0.2295 of AMDEA. The performance comparison of the algorithms in the ZDT5 and ZDT6 function tests is shown in Table 2.

**Table 2:** Performance comparison of algorithms in ZDT5 and ZDT6 function tests.

Test function	Algorithm	GD	IGD	HV
ZDT5	Improved MDEA	0.0563	0.2516	0.2617
	MDEA	0.0624	0.2745	0.2422
	AMDEA	0.0579	0.2539	0.2561
	NSGA-II	0.0678	0.2617	0.2416
ZDT6	Improved MDEA	0.0796	0.1901	0.2862
	MDEA	0.0802	0.1966	0.1761
	AMDEA	0.0799	0.1905	0.1936
	NSGA-II	0.0803	0.1924	0.1826

In Table 2, in the ZDT5 test, the improved MDEA outperforms the other algorithms in the IGD and HV metrics, which are 0.2516 and 0.2617, respectively. In the ZDT6 test, the improved MDEA has the highest value of HV, which is 0.2862. Moreover, the value of IGD is also smaller, which is 0.1901. The outcomes demonstrate that the enhanced MDEA performs well across the board in every test. The improved MDEA outperforms MDEA, AMDEA, and NSGA-II algorithms in measuring convergence, the degree of approaching the true optimal frontier, population uniformity, diversity, and other related indicators. Taken together, the improved MDEA shows superior performance advantages.

### 3.2. Analysis of the optimization results of the chpdeed system

The experiment further applies the improved MDEA to the CHPDEED model solution with time-varying multi-objective PSO (TV-MOPSO) algorithms NSGA-II-MOPSO and multi-objective monarch butterfly optimization (MOMVO) as comparisons. In this study, all comparison algorithms are executed within a uniform experimental environment. The parameters of each algorithm are optimized through a series of preliminary experiments. The convergence speed and the quality of solution of the algorithm on the test function are utilized as performance metrics. Table 3 displays the parameters for the algorithm.

**Table 3:** Algorithm parameter settings.

Parameter	Improved MDEA	TV-MOPSO	MOMVO	NSGA-II-MOPSO
Population size	200	200	200	200
Acceleration factor	/	0.5,2.0	/	1.5,1.7
Crossover probability	0.75	0.70	0.55	0.75
Scaling factor	[0.5,0.8]	/	0.65	/
Distribution index	/	/	20	/
Inertia weight	/	0.2,0.8	0.2,0.8	0.2,0.8

**Fig 10:**
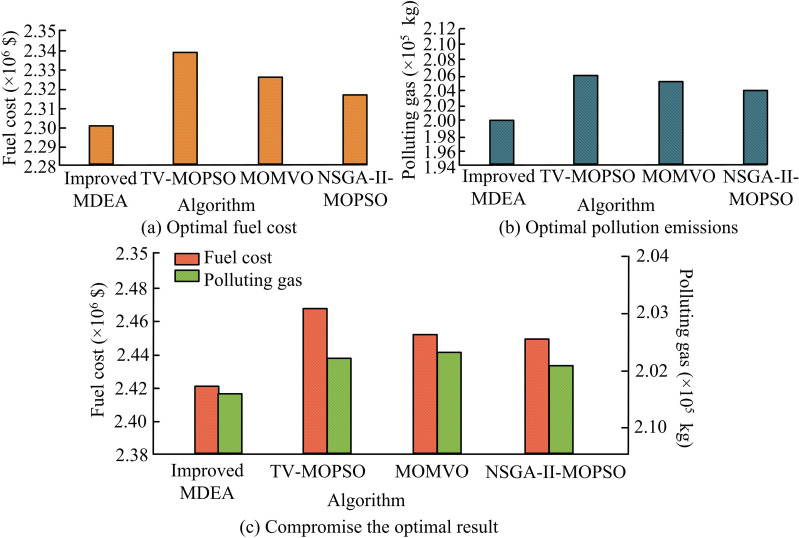
Optimization results of the CHPDEED system.

Based on an ieee 30-node system deployment, CHPDEED consists of two CHP units, seven generator units, and one generator unit with all-day power requirements in the range of [1120, 2100]MW and TP requirements in the range of [370, 470]MWth. In the peak period (18–22 o ‘clock), the power demand is high, close to 2100MW. Whereas, in the low period (0–6 o ‘clock), the power demand is low, about 1120MW. There is a certain power loss in the system, and its loss rate is about 3%−5%, which varies slightly in different periods due to load conditions. The schedule of each unit is as follows: The CHP unit runs all day and dynamically adjusts the power generation according to the TP demand and electrical load. Among the 7 generator sets, some units are based-load units, which operate stably throughout the day, and the rest are scheduled to start and stop according to load changes. The heating unit is mainly put into operation during the high TP demand period (18–22 o ‘clock) to meet the TP demand. The optimization results of the CHPDEED system are shown in Fig 10. To reach a compromise solution, it is necessary to collect comprehensive data related to fuel costs and pollution emissions, including price fluctuations of different fuels and quantitative indicators of various emissions. Then, a professional team composed of energy experts, environmental scholars, engineers, etc., is formed to use MOO algorithms to minimize fuel costs and minimize pollution emissions as OFs, while considering the actual constraints of system operation, including equipment performance limitations, energy supply stability, etc. Through repeated simulations and calculations, the balance between cost and emissions is explored and several candidate schemes are generated. These options are evaluated in detail, weighing the performance of each option in terms of cost savings and emissions reduction, and ultimately determining a compromise solution that can control fuel costs within an acceptable range while significantly reducing pollutant emissions, providing both economic and environmental benefits.

**Fig 11:**
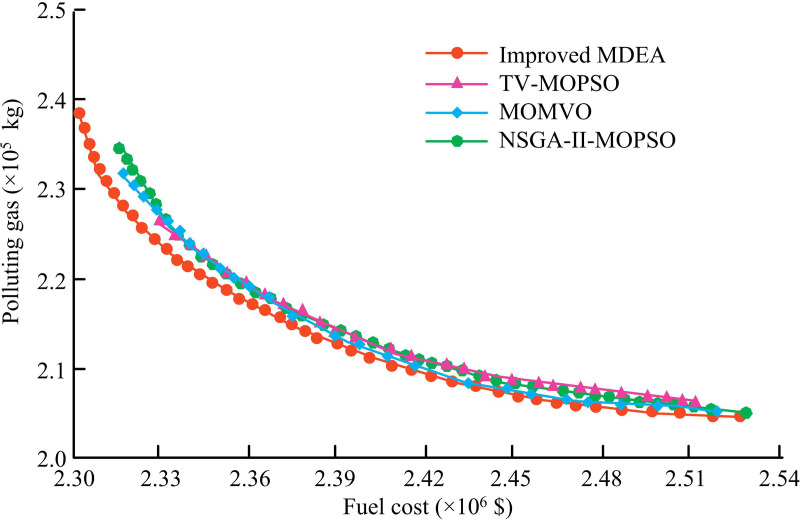
Comparison of POF for CHPDEED system.

Fig 10(a) shows the FC optimization results. The FC after improved MDEA, TV-MOPSO, MOMVO, and NSGA-II-MOPSO optimization are $2300590, $2339120, $2327031, and $2317135, respectively. Fig 10(b) shows the result of the optimization of pollution emission. The pollution emission after improved MDEA, TV-MOPSO, MOMVO calculation, and NSGA-II-MOPSO optimization are 200,285 kg, 205,862 kg, 205,247 kg, and 204,003 kg, respectively. In Fig 10(c), both fuel cost and pollution emission targets are considered in the selection of the best compromise. According to the optimal compromise result, the optimized fuel cost of the improved MDEA is $2421510 and the pollution emission is 201571 kg, both of which are lower than the optimized corresponding values of the other three algorithms. In practical applications, if more attention is paid to cost control, the fuel cost can be selected to be close to $23,590, and the pollution emission is relatively low at 200,285 kg. A greater focus on environmental benefits allows for the selection of pollution emissions close to 200,285 kg. While this results in an increase in fuel cost, it also ensures an acceptable range overall. Considering various factors, the compromise optimal solution obtained by the improved MDEA can achieve a good balance between the operating cost and performance of the system, which is a more ideal selection scheme and has obvious advantages over other comparative algorithms. The POF comparison for the CHPDEED system is shown in Fig 11.

**Fig 12:**
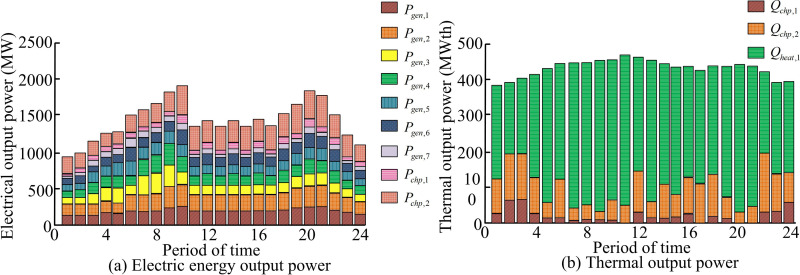
Histogram of system output power obtained by improving MDEA.

In Fig 11, in the comparison of the four algorithms, the POF distribution of the improved MDEA is better, with a larger range and its diversity is better. For the same FC spent, the improved MDEA corresponds to lower lower pollutant gas emissions. It demonstrates that, in comparison to the other four algorithms, the enhanced MDEA may identify a better solution throughout the optimization process, resulting in a system that performs better overall and strikes a better balance between operational cost and performance. The histogram of the system output power obtained by the improved MDEA is shown in Fig 12.

Fig 12(a) and 12(b) shows the histogram of electrical and thermal energy output power histogram. The electrical and thermal energy output power of the system are generally more stable during the 24 scheduling time periods. This indicates that the improved MDEA can effectively optimize the system operation, resulting in less power output fluctuation. The power distribution of each unit is more uniform, indicating that the improved MDEA has good balancing ability in different power outputs, ensuring stable and efficient operation of the system under multiple power output scenarios. The high power output period is continuous and stable, and there is no large power drop. This reflects that the improved MDEA is able to effectively avoid the power loss of the system during operation and enhance the overall performance and reliability of the system. Based on the optimization results of the improved MDEA for the CHPDEED system, the two CHP units run continuously throughout the day for 24 hours. Moreover, in the peak period of TP demand (18–22 o ‘clock), the CHP units give priority to meet the TP demand, and adjust the generation power according to the residual power load to maintain the power balance of the system. Among the 7 generator sets, 3 base-load units operate stably throughout the day to provide basic power supply. The remaining 4 units are scheduled to start and stop according to the change of power load in different periods. All of them are put into operation during the peak period of electricity consumption (18-22 o ‘clock). Furthermore, some units are shut down during the low period of electricity consumption (0-6 o ‘clock) to reduce energy consumption. The heating units are mainly put into operation from 16 to 22 o’clock. When the TP demand is high, it can effectively meet the TP demand. At other times, it starts and stops flexibly according to the actual heat load, ensuring that the system meets the heat demand while realizing the efficient use of energy. Table 4 displays a comparison of the algorithms’ performance metrics within the CHPDEED system.

**Table 4:** Comparison of performance metrics of algorithms in CHPDEED system.

Algorithm	HV			IGD		
Maximum	Minimum	Average	Maximum	Minimum	Average
Improved MDEA	0.0332	0.0310	0.0321	3581.3	721.5	2751.5
TV-MOPSO	0.0311	0.0305	0.0309	6251.6	2984.4	5298.4
MOMVO	0.0327	0.0293	0.0313	5248.3	983.8	3824.7
NSGA-II-MOPSO	0.0329	0.0301	0.0315	4937.2	837.8	3587.8

In Table 4, from the HV metrics, the MaxV of the improved MDEA is 0.032, the MinV is 0.030, and the average value is 0.0321, which are all higher than TV-MOPSO, MOMVO, and NSGA-II-MOPSO. In terms of IGD metrics, the mean value of improved MDEA is 2751.5, the MaxV is 3581.3, and the MinV is 721.5, all of which are smaller than the other compared algorithms. This suggests that the NDS set is nearer the actual optimal frontier and that the enhanced MDEA has greater convergence speed and variety in this system.

In conclusion, during the operation of the improved MDEA, the algorithm gradually converges as the number of iterations increases. In the initial stage, the particle distribution is relatively scattered, and the algorithm finds the potential optimal solution by constantly exploring the solution space. To illustrate this point, consider the 150-iteration case. Initially, the fit degree of the Pareto optimal front edge of the algorithm and the standard curve is low. However, as iterations progressed, the fit degree of the Pareto optimal front edge and the standard curve of the improved MDEA exhibited a marked improvement. Concurrently, the particle distribution uniformity underwent a gradual enhancement. As the number of iterations increases to 300, this fit continues to improve, indicating that the algorithm is continuously optimized during the iteration process and gradually converges to the solution set closer to the true Pareto optimal front. In the CHPDEED system optimization process, the improved MDEA also gradually approaches the better value as the number of iterations increases, such as fuel cost and pollutant emission. It can be concluded from the comparison of optimization results of different algorithms in Fig 10 and POF comparison in Fig 11. The improved MDEA converges to a better solution space faster and is superior to other comparative algorithms in convergence speed and convergence quality.

To verify the scalability of the improved MDEA, the study applied it to the deployment of a large-scale CHPDEED system based on an ieee 30-node system, including 20 CHP units, 80 generator units, and 10 heating units, with a full-day power demand range of [10360, 21500]MW. TP requirements range from [3900, 4800]MWth. At the same time, the algorithms in references [16,17], and [18] are compared with the improved MDEA. The results of application in large-scale systems are shown in Table 5.

**Table 5:** Comparison of application results in large-scale systems.

Algorithm	Optimal fuel cost		Optimal pollution discharge		Compromise optimal solution	
Fuel cost ($)	Pollution emission (kg)	Fuel cost ($)	Pollution emission (kg)	Fuel cost ($)	Pollution emission (kg)
Improved MDEA	24673815	2538482	25861728	2239172	25087314	2298731
Literature [16]	26752813	2526813	27561384	2432817	26682735	2489637
Literature [17]	26458315	2435827	27518310	2402712	26678234	2423715
Literature [18]	25152538	2332589	25446816	2282124	25184951	2314514

The results in Table 5 show that the improved MDEA has performance in fuel cost optimization, pollution emission optimization, and compromise optimal solution. Compared with the reported results published in literatures [16,17], and [18], the validity and reliability of these application results are further verified, providing a valuable reference for relevant decision-making in large-scale systems. To verify the statistical significance of the experimental results, *t* test is used to evaluate the differences. The statistical test results are shown in Table 6.

**Table 6:** Statistical test result.

Comparative content	Algorithm 1	Algorithm 2	*t*	*p* value
ZDT1 function GD indicator	Improved MDEA	MDEA	2.14	0.036
ZDT1 function IGD indicator	Improved MDEA	MDEA	2.05	0.044
ZDT2 function HV index	Improved MDEA	MDEA	1.98	0.052
CHPDEED indicates the system HV indicator	Improved MDEA	TV-MOPSO	2.31	0.024
CHPDEED indicates the IGD of the system	Improved MDEA	AMDEA	12.15	0.000

In the comparison of the GD index of the ZDT1 function, the *p* value of the improved MDEA is 0.036 and less than 0.05 compared with the value of the assumed MDEA, and the difference is statistically significant, indicating that the improved MDEA performs better on this index. For the IGD index of the ZDT1 function, the *p* value of the improved MDEA compared with the hypothetical AMDEA is about 0.044 less than 0.05. It indicates that the performance of the improved MDEA on this index is significantly different from that of the comparison algorithm. In the comparison of HV index of ZDT2 function, the *p* value is about 0.052 and greater than 0.05. It means that the improved MDEA and NSGA-II algorithm have no significant difference in this index. In the comparison of HV index of CHPDEED system compared with TV-MOPSO algorithm, the *p* value of the improved MDEA is about 0.024 and less than 0.05. It indicates that the advantage of the improved MDEA in this index is statistically significant. For the IGD index of the CHPDEED system, the *p* value of the improved MDEA is much less than 0.001 compared with the AMDEA, and the difference is extremely significant. It further reflects the good performance of the improved MDEA on this index.

## 4. Discussion and conclusion

Focusing on the MOO challenges in engineering, the research aimed at exploring efficient algorithms applicable to CHPDEED to achieve quality trade-offs between economic costs and pollutant emissions. The research designed dynamic scaling factor and adaptive crossover probability. Combining the non-dominated ordering and congestion distance calculation, it added a quadratic variation link, and propose an improved MDEA. It constructed a CHPDEED model by combining various power constraints covering FC, pollution emission, and various types of power constraints. The outcomes indicated that the POF of the improved MDEA fitted the standard curve better than the comparison algorithms with a uniform distribution at 150 iterations. Moreover, the advantage was even more obvious at 300 iterations, as tested by the set of ZDT test functions. In the ZDT1 test, the GD of the improved MDEA was as low as 0.0513, the IGD was 0.3265, and the HV was up to 0.1301. It outperformed the MDEA, AMDEA, and NSGA-II algorithms under multiple test functions. Applied to CHPDEED system, the FC was optimized to $2300590 and the pollution emission was reduced to 200285 kg. The cost of the compromise solution $2421510 and the emission 201571 kg were ahead of similar algorithms. The Pareto front distribution of the improved MDEA was excellent and wide-ranging, and the system power output was stable, with little fluctuation of electrical and thermal energy, and the power of each unit was balanced. The performance index of the improved MDEA reached 0.0321 for HV and 2751.5 for IGD, showing good convergence and diverse characteristics. The study shows that the improved MDEA and CHPDEED models are highly effective in accurately controlling the economic and emission trade-offs of the CHP system, ensuring stable operation, and providing new ideas for similar MOO and energy system scheduling. When applying the improved MDEA to large-scale systems, the following considerations are made in terms of computational efficiency. First, the algorithm adopts an adaptive parameter adjustment strategy to reduce unnecessary search steps and improve search efficiency. In large-scale systems, the search space experiences a precipitous increase with the rise in decision variables. The adaptive scaling factor and crossover probability can expedite the algorithm’s identification of the optimal solution region and mitigate the computational complexity. Conversely, parallel computing technology is employed in the implementation of the algorithm. The multiprocessing library is utilized to parallelize the population evolution process, thereby leveraging the advantages of multi-core processors and reducing computing time. However, there are still limitations in the study. Although the parameters of the algorithm are adaptive, the initial parameter setting still relies on experience, and the generalizability of the algorithm should be improved. The algorithm’s ability to respond in real time and adjust dynamically in the face of sudden changes in complex operating conditions, such as sudden failures of the CHP unit or huge shocks in the energy market, is not good enough. The model only initially considers common constraints, without digging into the details of unit aging and energy transmission loss, which is far from the complex reality. In the future, the research will deepen the algorithm theory, integrate reinforcement learning and other technologies to realize intelligent parameter optimization and broaden the application scenarios. Then the CHP model will be refined to incorporate more uncertainty factors, simulate extreme working conditions, and optimize the algorithmic decision-making with big data, so as to promote the efficient, green and intelligent development of the CHP system. At the same time, future research plans to incorporate more real-world constraints to improve the applicability of the algorithm to complex real-world scenarios. In regard to the issue of demand uncertainty, a stochastic process or probabilistic model is employed to characterize the variability in electricity and heat load demand. This approach enables the algorithm to identify the optimal scheduling strategy under uncertain conditions. For the unit failure rate, the unit failure probability model is established and integrated into the scheduling model to ensure that the economic and environmental benefits of the system can be optimized under the condition that the unit may fail. In light of the volatile nature of fuel prices, the fuel market price information is obtained in real time. This information is then incorporated into the model, thereby allowing the algorithm to adjust the scheduling scheme in a timely manner in response to market fluctuations. The objective of this adjustment is to achieve the optimal scheduling of the system.
